# Dysphagia Lusoria: A Little Known Cause of Chest Pain

**DOI:** 10.7759/cureus.20085

**Published:** 2021-12-01

**Authors:** Zaka Ahmed, Ndausung Udongwo, Safa Albustani, Sobaan Taj, Kyle Wiseman, Halah Alchalabi, Mohammad A Hossain

**Affiliations:** 1 Internal Medicine, Jersey Shore University Medical Center, Neptune City, USA; 2 Medicine, Hackensack Meridian School of Medicine, Nutley, USA

**Keywords:** vascular, aberrant, lusoria, subclavian artery, dysphagia

## Abstract

Dysphagia lusoria is a congenital abnormality characterized by an aberrant right subclavian artery. It often presents as either an incidental finding on imaging or chronic dysphagia. We describe the case of a 66-year-old female who presented with severe chest pain, worse with swallowing, along with an ongoing globus sensation. She was found to have a negative cardiac workup for ischemia with a subsequent computed tomography angiogram (CTA) of the chest showing an abnormal right subclavian artery. We emphasize the unique diagnostic approach of this rare anatomical anomaly and its potential presentation that worsens with deglutition.

## Introduction

Dysphagia lusoria (DL) is an uncommon congenital abnormality caused by an abnormal or twisted right subclavian artery. Depending on the anomaly, the patient usually complains of difficulties swallowing. Dysphagia lusoria is caused by an aberrant origin of the subclavian artery, which travels posterior to the esophagus, causing compressive sensations [1]. In these individuals, the presence of an aneurysm or substantial occlusive atherosclerosis may contribute to an earlier clinical presentation [2]. Our team presents the case of a 66-year-old female who arrived at the emergency department (ED) complaining of chest discomfort and difficulty in swallowing solids. Based on our review of the literature, chest pain is a very rare presenting symptom for patients with DL.

## Case presentation

A 66-year-old female with a past medical history of hypertension, diabetes mellitus, benign thyroid nodule, anxiety, gastroesophageal reflux disease, and hiatal hernia presented to the ED with complaints of chest discomfort for the past week. The chest pain was rated nine out of 10 in severity, radiated to the back/left arm, and was precipitated by swallowing. The patient endorsed a globus sensation as well but denied any fever, chills, cough, shortness of breath, nausea, vomiting, weight loss, recent trauma, recent travel history, or sick contacts. Family history was non-contributory. She denied any tobacco or illicit drug use in the past and only drank alcohol occasionally. Her home medications included glipizide, 10 mg once daily, metformin, 1,000 mg twice daily, atenolol, 100 mg once daily, alprazolam, 0.25 mg twice daily as needed, and pantoprazole, 40 mg three times weekly. 

Initial vitals revealed a blood pressure of 214/93 mm Hg, heart rate of 66 beats per minute, respiratory rate of 13 breaths per minute, a temperature of 97.8 °F, and oxygen saturation of 98% on room air. On physical examination, she was not in any acute distress. The cardiopulmonary assessment was unremarkable, and the chest pain was not reproducible on the physical examination. Throat examination did not reveal any erythematous changes or edema. Laboratory analysis was unremarkable, except for a mildly elevated troponin level, as shown in Table 1.

**Table 1:** Laboratory Values

Laboratory study	Results	References
Hemoglobin (g/dL)	12.7 g/dL	12.0 - 16.0 (g/dL)
White blood cells (10*3/µL)	4.3 10*3/µL	4.5 - 11.0 (10*3/µL)
Glucose (mg/dL)	155 mg/dL	70 - 99 (mg/dL)
Blood urea nitrogen (BUN) (mg/dL)	4.0 mg/dL	5 - 25 (mg/dL)
Sodium (mmol/L)	138 mmol/L	136 - 146 (mmol/L)
Potassium (mmol/L)	3.4 mmol/L	3.5 - 5.0 (mmol/L)
Chloride (mmol/L)	102 mmol/L	96 - 110 (mmol/L)
Calcium (mg/dL)	9.2 mg/dL	8.5 - 10.5 (mg/dl)
Magnesium (mg/dL)	1.9 mg/dL	1.3 - 2.5 (mg/dL)
Bicarbonate (mmol/L)	27 mmol/L	24 - 31 (mmol/L)
Creatinine (mg/dL)	0.78 (mg/dL)	0.44 - 1.00 (mg/L)
Anion gap (mmol/L)	9 (mmol/L)	5 - 13 (mmol/L)
Troponin (ng/L)	0.06 (ng/mL)	< 0.04 (ng/mL)

**Figure 1:**
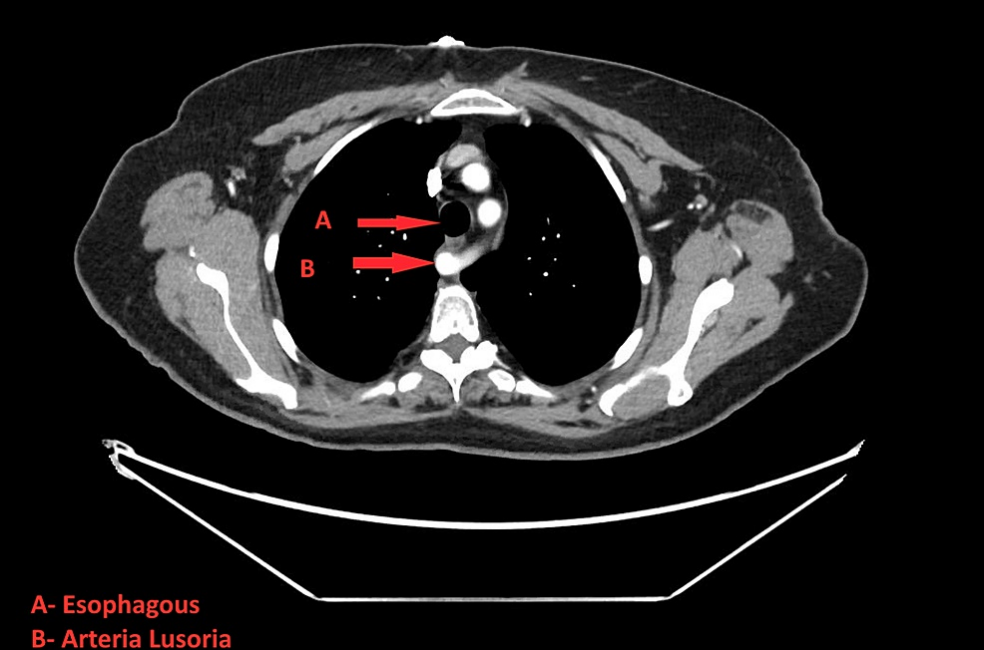
Computed tomography angiogram of the chest, abdomen, and pelvis The arrows above point to an aberrant right subclavian artery that lies posterior to the esophagus at the level of the upper thorax.

**Figure 2:**
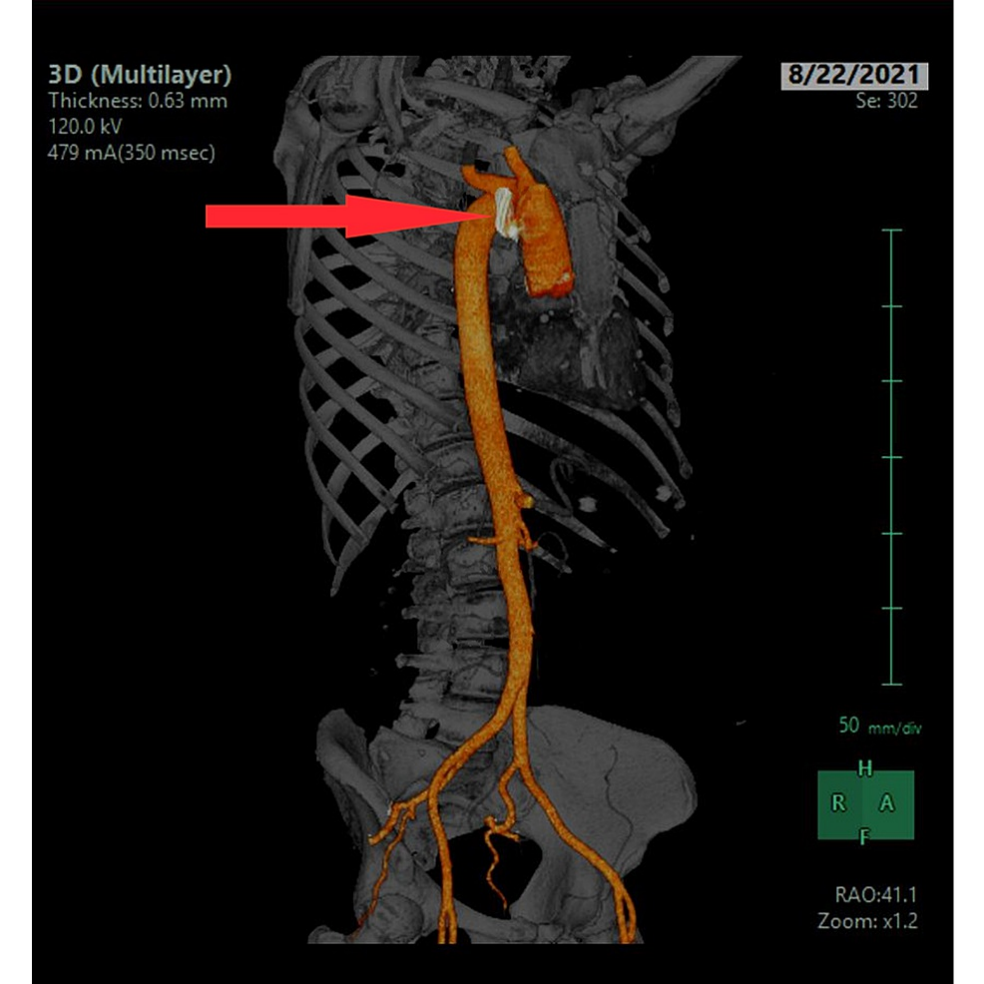
Computed tomography angiogram of the chest, abdomen, and pelvis The red arrow is pointing towards the lesion.

**Figure 3:**
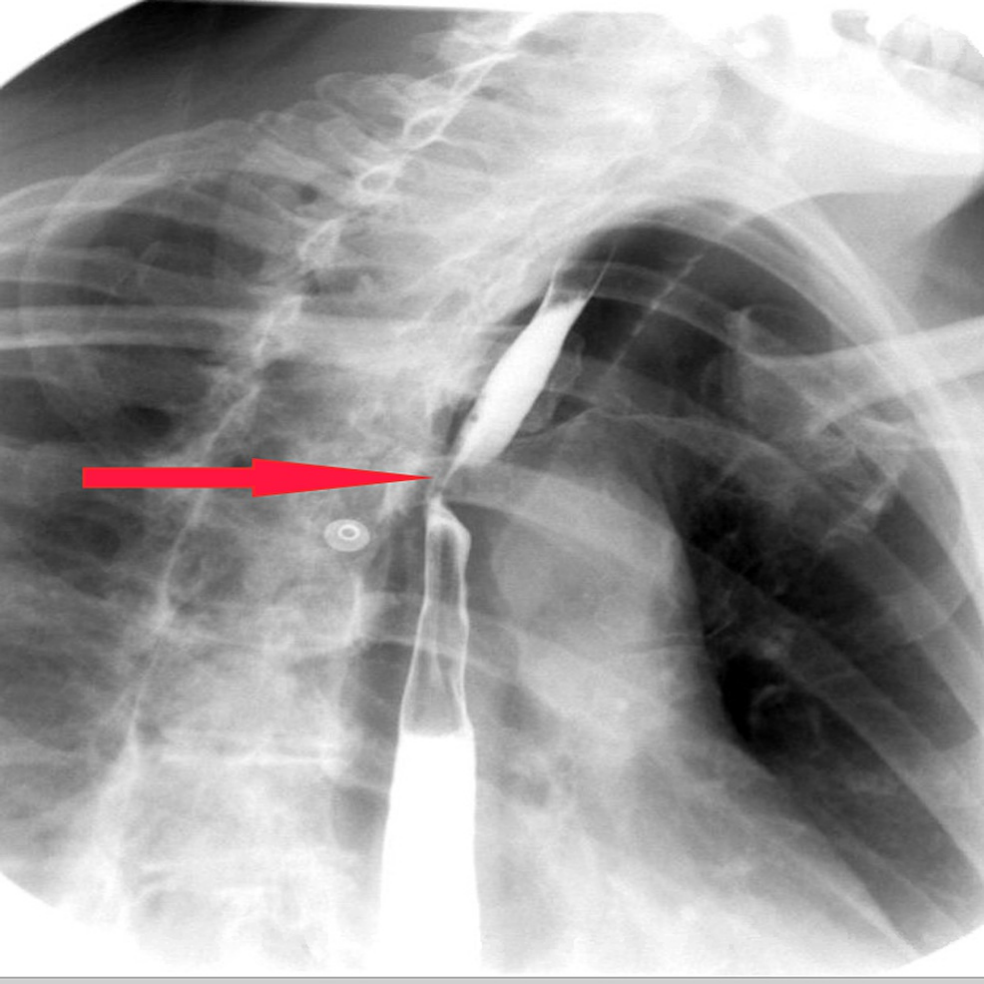
Mild smooth narrowing of the esophagus, at the level of aortic arch, on the standing views which does not persist on the right anterior oblique (RAO) imaging series

Electrocardiogram (EKG) revealed a normal sinus rhythm at a rate of 72 beats per minute with no ST or T wave changes. Chest x-ray was unremarkable. Treadmill exercise nuclear stress test (NST) and echocardiographic findings were unremarkable. Computed tomography angiogram (CTA) of the chest revealed an abnormal right subclavian artery lying posterior to the esophagus at the level of the upper thorax (Figures 1, 2). Image findings from a single-contrast esophagram x-ray revealed some mild, transient, smooth narrowing of the barium-filled esophagus at the level of the aortic arch (Figure 3).

She was diagnosed with hypertensive urgency and placed on a nitroglycerin drip. A low-intensity heparin drip was also started for a presumptive diagnosis of non-ST segment elevation myocardial infarction (NSTEMI), type II. Her blood pressure stabilized in less than six hours. 

Vascular surgery, gastroenterology, and cardiology were among the specialists on board. Dysphagia lusoria was diagnosed based on results from CTA of the chest and the esophagram. Upon evaluation by vascular surgery, no surgical intervention was pursued due to the high risks associated with the procedure. With a normal NST and echocardiogram, the patient was cleared by cardiology for discharge. She was counseled on proper mastication of food, maintenance of an upright position for at least three hours after meals, and intake of protein shakes to maintain body weight. She was discharged on pantoprazole, 40 mg daily, and baclofen, 5 mg three times a day, and advised to follow up with a gastroenterologist to assess and manage future dietary modifications.

## Discussion

In terms of the embryonic aortic arch, the most common defect found in the general population is an anomalous right subclavian artery (ARSA). [1]. ARSA is caused by a persistent seventh intersegmental artery and involution of the fourth vascular arch within the right dorsal aorta [2]. In most cases, the aberrant artery runs posterior to the esophagus. In a small proportion of cases, it runs anterior to the esophagus, as described in Bayford's initial report of the condition [3]. ARSA is sometimes accompanied by a wide base known as Kommerell's diverticulum [1]. 

In rare cases, Kommerell's diverticulum may develop an aneurysm, resulting in esophageal compression and dysphagia [4-5]. The bulk of the available research estimates that the frequency of an anomalous subclavian artery in the general population is approximately 0.5% to 1.8% [6]. The majority of these lesions are caused by a right subclavian artery branching from the left aortic arch. A similar anomaly can arise as the result of a left-sided aberrant subclavian artery connecting to a right-sided aortic arch; however, this vascular abnormality is far less common [7-8]. When the right dorsal artery remains patent and either the left 4th arch or the left dorsal aorta regress abnormally, this abnormality occurs. When the abnormality is coupled with a left-sided ductus arteriosus connecting from the left subclavian artery to the proximal left pulmonary artery, a full vascular ring is created. In our literature review, we found that the presenting symptom in 91% (31/ 34) of reported cases was dysphagia [1].

Dynamic barium swallow tests, which include the evaluation of a solid bolus swallow, can be used to screen for dysphagia lusoria. To identify the vascular lesion and arrange surgical treatments, a CT scan of the chest or magnetic resonance imaging with vascular reconstruction are utilized. Manometry can reveal a variety of problems and is thus not useful in the diagnosis. Janssen et al. conducted manometric studies on six patients with dysphagia lusoria and found abnormalities in five of the six patients, with two studies revealing diminished amplitude contractions, two revealing a high-pressure zone (increase in intrabolus pressure) above the aberrant artery, and one revealing a hypo-contractile zone proximal to the aberrant artery [9]. When dysphagia arises as a result of aging, it appears likely that non-specific age-related manometric anomalies, such as an increased prevalence of peristaltic failure, may be contributing to the dysphagia in these individuals [10-11].

There are several causes for reported late-onset symptoms. One theory is that motility problems and esophageal stiffness arise as a result of aging [9, 12]. Atherosclerosis-induced vascular alterations leading to stiffness of the obstructing artery, aortic elongation with greater traction on the obstructing artery, or aneurysmal dilatation in the context of Kommerell's diverticulum have all been postulated as alternative causes [9, 12].

The therapy for patients with dysphagia lusoria is determined by the severity of the symptoms and their influence on the patient’s ability to maintain their weight and nutrition. It appears that around half of the patients’ symptoms may be controlled via dietary changes, eating slowly, and chewing thoroughly, like in our patient. Severe symptoms that do not respond to dietary approach and swallowing technique modification may require surgical therapy [12]. Gross was the first to describe the surgical treatment of this disease, reporting the division and closure of an anomalous right subclavian artery by left thoracotomy in a four-month-old newborn [13].

## Conclusions

Dysphagia is a common symptom seen in daily clinical practice and is often overlooked. For patients who present with chest pain and dysphagia, further clinical history and early evaluation are critical for diagnosis and appropriate management. Although dysphagia lusoria is rare, clinicians should be aware of this uncommon congenital abnormality. This case demonstrates the significance of early imaging by barium swallow to assess for esophageal dysphagia and a CT chest angiography to identify an aberrant subclavian artery origin with esophageal compression.

## References

[1] Levitt B, Richter JE (2007). Dysphagia lusoria: a comprehensive review. Dis Esophagus.

[2] Taylor M, Harris KA, Casson AG, DeRose G, Jamieson WG (1996). Dysphagia lusoria: extrathoracic surgical management. Can J Surg.

[3] Asherson N (1979). David Bayford. His syndrome and sign of dysphagia lusoria. Ann R Coll Surg Engl.

[4] Triantopoulou C, Ioannidis I, Komitopoulos N, Papailiou J (2005). Aneurysm of aberrant right subclavian artery causing dysphagia lusoria in an elderly patient. AJR Am J Roentgenol.

[5] Singh S, Grewal PD, Symons J, Ahmed A, Khosla S, Arora R (2008). Adult-onset dysphagia lusoria secondary to a dissecting aberrant right subclavian artery associated with type B acute aortic dissection. Can J Cardiol.

[6] Richardson JV, Doty DB, Rossi NP, Ehrenhaft JL (1981). Operation for aortic arch anomalies. Ann Thorac Surg.

[7] McNally PR, Rak KM (1992). Dysphagia lusoria caused by persistent right aortic arch with aberrant left subclavian artery and diverticulum of Kommerell. Dig Dis Sci.

[8] Panduranga P, Al-Delamie T, Ratnam L, Al-Mukhaini M, Zachariah S (2011). Repair of Kommerell's diverticulum with aberrant left subclavian artery in an elderly patient with right aortic arch and dysphagia lusoria. J Card Surg.

[9] Janssen M, Baggen MG, Veen HF, Smout AJ, Bekkers JA, Jonkman JG, Ouwendijk RJ (2000). Dysphagia lusoria: clinical aspects, manometric findings, diagnosis, and therapy. Am J Gastroenterol.

[10] Grishaw EK, Ott DJ, Frederick MG, Gelfand DW, Chen MY (1996). Functional abnormalities of the esophagus: a prospective analysis of radiographic findings relative to age and symptoms. AJR Am J Roentgenol.

[11] Tack J, Vantrappen G (1997). The aging oesophagus. Gut.

[12] Bennett AL, Cock C, Heddle R, Morcom RK (2013). Dysphagia lusoria: a late onset presentation. World J Gastroenterol.

[13] Gross RE (1946). Surgical treatment for dysphagia lusoria. Ann Surg.

